# Serum Lipoprotein (a) Levels in Black South African Type 2 Diabetes Mellitus Patients

**DOI:** 10.1155/2016/5743838

**Published:** 2016-10-19

**Authors:** Jim Joseph, Farzana Ganjifrockwala, Grace George

**Affiliations:** Division of Medical Biochemistry, Department of Human Biology, Faculty of Health Sciences, Walter Sisulu University, Mthatha Campus, Nelson Mandela Drive, Mthatha 5100, South Africa

## Abstract

Lipoprotein (a) (Lp(a)) which is a low-density lipoprotein-like particle containing apo(a) is considered as an emergent cardiovascular risk factor. Type 2 diabetes mellitus (T2DM) is associated with a two- to threefold increase in the risk of cardiovascular disease (CVD). The aim of this study was to investigate the levels of Lp(a) in Black South African T2DM patients and its association with other metabolic factors. 67 T2DM patients and 48 healthy control participants were recruited for the cross-sectional study. The Lp(a) level was determined by ELISA and the result was analyzed using SPSS. The Lp(a) level in diabetics was found to be significantly increased (*P* = 0.001) when compared to the normal healthy group. In the diabetic group, the Lp(a) levels correlated significantly with the duration of diabetes (*P* = 0.008) and oxidized LDL (ox-LDL) levels (*P* = 0.03) and decreased total antioxidant capacity (*P* = 0.001). The third tertile of Lp(a) was significantly correlated with increased ox-LDL, C-reactive protein, and triglycerides and decreased total antioxidant capacity.

## 1. Introduction

Type 2 diabetes mellitus (T2DM) is a multifactorial metabolic disorder characterized by chronic hyperglycemia caused by decreased production or sensitivity to insulin [1]. Chronic hyperglycemia results in a number of complications including cardiovascular disease (CVD). Diabetic patients have more than double the risk of CVD-related mortality when compared to age-matched controls [2]. The traditional cardiovascular risk factors associated with diabetes are not sufficient to explain the high rate of cardiovascular incidents in diabetic patients. Lipoprotein (a) (Lp(a)) is an emerging cardiovascular risk factor which is also associated with diabetes [3, 4]. Lp(a) is a low-density lipoprotein (LDL) particle with the glycoprotein apo(a) covalently bound to apo B-100 [5]. Apo(a) is a highly glycosylated hydrophilic apolipoprotein which is synthesized by the liver. Apo(a) is structurally homologous to the plasma protein plasminogen which is involved in the lysis of clots [6]. Like plasminogen, apo(a) is composed of two kringle domains and a serine protease domain. The size of one of the domains, kringle IV type 2, exhibits a wide variation and hence the size of apo(a) is heterogeneous among the general population [7]. The concentration of Lp(a) in plasma depends on the isoform of kringle IV type 2 which is genetically determined. The size of the apo(a) isoform inversely correlates with its plasma concentration and accounts for about 40% of its variation in plasma concentration [8]. Apo(a) by its similarity to plasminogen can competitively inhibit the functioning of this zymogen and hence increase the risk of atherosclerotic vascular disease. The similarity of Lp(a) to LDL and its ability to undergo oxidation are another reason why it has been implicated in atheroma development and has been suggested to be involved in foam cell formation, smooth cell proliferation, endothelial dysfunction, and vascular inflammation [9, 10]. Several epidemiological studies have found clear association between Lp(a) and CVD and have suggested Lp(a) to be an independent risk factor for CVD [11, 12]. Guyton et al. observed racial differences in mean Lp(a) levels. In their study, they found that Black participants had almost double the amount of mean Lp(a) levels compared to Whites [13]. Differences in mean Lp(a) values among different ethnic groups were also noted by Sandholzer et al. [14]. Early studies on Lp(a) levels among Blacks concluded that higher Lp(a) levels did not contribute to any increased cardiovascular risk among the Black population [13, 15, 16]. However, Virani et al. in a 20-year follow-up study have shown that high Lp(a) levels are equally and positively associated with the incidence of CVD in both Blacks and Whites [17].

The relation between Lp(a) and T2DM is still not clear with some studies showing a higher level of Lp(a) in diabetics [18, 19] and some showing a decreased level or no difference between diabetics and controls [20, 21]. In African studies, increased Lp(a) has generally been observed in T2DM patients [22, 23].

## 2. Material and Methods

This cross-sectional study was approved by the Health Research Ethics and Bio-Safety Committee of Walter Sisulu University (WSU) (protocol number 013/012) and was carried out at the Medical Biochemistry Laboratory, WSU, Mthatha. A total of 115 participants (48 controls and 67 patients with type 2 diabetes) were recruited for the study. The mean age of the controls was 54.7 ± 6.1 and that of the diabetic patients was 55.9 ± 5.5. The number of male participants was 40 (15 controls and 25 diabetics), and females were 75 (33 controls and 42 diabetics). Participants with a positive clinical history of T2DM were approached. The participants were excluded if they had any other chronic diseases apart from diabetes and hypertension. Participants with known diabetic complications (micro- and macrovascular complications) and those on insulin treatment and lipid-lowering medication were also excluded. Healthy control participants were selected from the local population. The participants were included in the study following informed consent. Interviewer administered questionnaires were used to obtain information on the duration of diabetes, medication, and lifestyle. The blood pressure and anthropometric measurements of all participants were also taken. The body mass index (BMI) was calculated as the weight (kg)/height (m)^2^. Blood and urine samples were collected from the participants following overnight fast into appropriate vacutainer tubes and sterile urine containers. The serum or plasma was removed within an hour and refrigerated at −80°C for analysis. Apart from a comprehensive metabolic panel, the samples were also used for analyses of urinary albumin and creatinine, insulin, and C-reactive protein (CRP), all of which were performed at the National Health Laboratory Services (NHLS) using the Roche Cobas 6000 autoanalyzer. Following evaluation of the results, participants with signs of liver disease, renal disease, and infection were excluded. Serum samples which were stored at −80°C were used in the determination of the Lp(a), total antioxidant (TAO) capacity, and oxidized LDL (ox-LDL) concentration, within a month. The serum TAO capacity was measured by a commercial kit from Sigma-Aldrich using the ABTS method. Lp(a) and ox-LDL were determined using sandwich ELISA kits from Mercodia (Uppsala, Sweden). The absorbance was read using a Biotek analyzer at 450 nm. The ox-LDL assay uses murine monoclonal antibody mAb-4E6 directed against oxidized apo B, while the Lp(a) assay uses antibodies directed against epitopes of apo(a).

The data was analyzed statistically using the IBM Statistical Package for the Social Sciences (SPSS Inc., Chicago, IL, USA, version 16). Shapiro-Wilk test was used to check for normality of the data. Normally distributed data was expressed as mean ± standard deviation (SD) and the independent sample *t*-test was done to check for the difference between groups. Data which did not exhibit normal distribution was expressed as median and interquartile range and the difference between groups was determined by Mann–Whitney *U* test. Participants were divided into tertiles according to their serum Lp(a) levels for correlation analysis: tertile 1 (*n* = 19), Lp(a) < 320 U/L; tertile 2 (*n* = 18), Lp(a) = 320–728 U/L; and tertile 3 (*n* = 30), Lp(a) > 728 U/L. Spearman's correlation tests were performed to analyze the correlation between the variables within the various groups. Kruskal-Wallis test was used to check correlations between other variables and Lp(a) across the tertiles. *P* < 0.05 was considered as statistically significant.

## 3. Results 

The mean age of the controls was 54.7 ± 6.1 and that of the diabetic patients was 55.9 ± 5.5. The mean duration of diabetes was 7.5 ± 6.5 years. The difference in total cholesterol and LDL values between the two groups did not show any significance. The triglycerides (TG) were significantly higher in the diabetic group (*P* = 0.007) while the high-density lipoprotein (HDL) level was significantly lower in the diabetic group (*P* = 0.041). Some of the metabolic characteristics of the participants are summarized in Table 1.

The Lp(a) was significantly higher in the diabetic group when compared to the normal (control) group as shown in Table 2.

The Lp(a) levels in the healthy controls were higher in females, 404 (274–550) U/L, than in males, 297 (135–622) U/L; however, this difference was not statistically significant.

No significant correlations were observed between Lp(a) levels and other parameters in the control population. In the diabetic population, the Lp(a) levels correlated significantly with ox-LDL (*r* = 0.26, *P* = 0.03) and LDL cholesterol concentration (*r* = 0.266, *P* = 0.026). Lp(a) levels were also significantly correlated to duration of diabetes (*r* = 0.31, *P* = 0.008). There was a negative correlation between Lp(a) and TAO capacity (*r* = −0.223, *P* = 0.026).

The ox-LDL and CRP levels increased across the tertiles of Lp(a), while the TAO capacity decreased as shown in Table 3. The correlation between Lp(a) levels and other variables in each Lp(a) tertile is summarized in Table 4.

## 4. Discussion

This study revealed that there was a significantly higher level of Lp(a) among the T2DM patients when compared to normal people. Several researchers have reported similar findings in T2DM patients [18, 19, 24]. Studies on Lp(a) levels in Africa have also reported elevated levels in the T2DM population. Mohieldein et al. in 2014 reported that T2DM patients in Sudan had a significantly higher Lp(a) level [22]. This was similar to the finding of Ogbera and Azenabor in Nigeria [23]. The high level of Lp(a) in diabetics can be because of the decreased clearance of Lp(a) from the serum due to glycation of its apolipoproteins [25]. Kadkhodaei et al. in 2006 stated that the glycated Lp(a) in the diabetic population is significantly higher in the diabetic population [26]. In this study, the patients with longer duration of diabetes had a higher level of Lp(a). This is similar to the findings of Chandni and Ramamoorthy [27]. The relationship between Lp(a) levels and duration of diabetes is clinically important because the duration of diabetes confers a twofold increase in the risk of vascular complications [28].

The Lp(a) levels in the diabetic population correlated significantly with the LDL cholesterol levels in this study. This correlation has been observed in other studies also [28, 29]. This correlation can be attributed to the contribution of the Lp(a) cholesterol levels in the calculation of LDL cholesterol using the Friedewald formula. Lp(a) has been shown to be independent of or weakly associated with the concentrations of other lipoproteins in other studies [29–31].

The Lp(a) levels showed a significant correlation with the amount of ox-LDL in the diabetic group. This correlation between Lp(a) and ox-LDL was not observed among the controls. In diabetics, there is increased oxidation of lipoproteins [32, 33] including Lp(a) because it is as prone as LDL to oxidation [34, 35]. Moreover,* in vitro* studies have shown the preferential transfer of oxidized phospholipids from ox-LDL to Lp(a) thereby increasing the oxidation of Lp(a) [36, 37]. The oxidatively modified Lp(a) can cross-react with the antibodies used in the ox-LDL analysis.

The diabetic patients had increased levels of inflammation as shown by the increased CRP levels. The diabetic patients also had increased oxidative stress as evidenced by the increased ox-LDL levels and decreased TAO capacity. Bergmark et al. have stated that increased levels of oxidized phospholipids observed during inflammation and oxidative stress may cause the overexpression of Lp(a). The increased Lp(a) can bind and transport the oxidized phospholipids thereby ameliorating the inflammation and oxidative stress. However, in high concentrations, this beneficial Lp(a) molecule can become proinflammatory and atherogenic [37]. This is similar to our observation among diabetic individuals: a negative correlation was observed between CRP and the first two tertiles of Lp(a). However, the third tertile of Lp(a) was positively and significantly correlated to CRP, TG, and ox-LDL levels. Increased levels of ox-LDL and Lp(a) have been found to be independently associated with a poor prognosis following acute myocardial infarction [38]. Hence, the increase in both ox-LDL and Lp(a) among the diabetics in this study not only exposes them to an increased risk of a cardiovascular event but also can negatively impact the prognosis of these patients following such an event.

## 5. Conclusion

T2DM is associated with increased levels of various cardiovascular risk factors including Lp(a) and ox-LDL. Very high levels of Lp(a) among diabetics are proinflammatory.

## Figures and Tables

**Table 1:** Clinical and metabolic characteristics of type 2 diabetics and healthy controls.

Variable	Control	Diabetic	*P* values
BMI (kg/m^2^)	28.1 ± 6.2	31.4 ± 7	0.03^*∗*^
Waist-hip ratio	0.87 (0.85–0.89)	0.92 (0.87–0.94)	<0.01^*∗*^
Total cholesterol (mmol/L)	4.8 (3.9–5.4)	4.9 (3.9–5.3)	0.99
HDL (mmol/L)	1.4 (1.1–1.6)	1.2 (1.0–1.4)	0.03^*∗*^
Triglycerides (mmol/L)	1.1 (0.9–1.5)	1.3 (1.1–1.6)	0.02^*∗*^
LDL (mmol/L)	2.8 (2.2–3.2)	2.9 (2.0–3.4)	0.68
CRP (mg/L)	1.7 (1.0–4.2)	4.0 (2.3–7.7)	0.01^*∗*^
TAO capacity (mM)	0.6 ± 0.23	0.5 ± 0.24	0.001^*∗*^
Oxidized LDL (U/L)	73 (51.2–90)	110 (80.7–142.3)	<0.001^*∗*^

Data is shown as mean ± SD (standard deviation) or median (interquartile range). The mean difference is significant at ^*∗*^ *P* < 0.05. BMI: body mass index; HDL: high-density lipoprotein; LDL: low-density lipoprotein; CRP: C-reactive protein.

**Table 2:** Median Lp(a) between the groups.

Variable	Normal	Diabetic	*P* value
Lp(a) (U/L)	329 (178–474)	670 (314–1331)	0.001^*∗*^

Data is shown as median and IQR (interquartile range). The median difference is significant at ^*∗*^ *P* < 0.05.

**Table 3:** Clinical characteristics of diabetic patients by Lp(a) tertiles.

	Tertile 1	Tertile 2	Tertile 3	*P* value
Age	46.4 ± 9.5	47.3 ± 8.2	51.8 ± 8.6	0.051
Diabetic duration	1.7 ± 2.9	4.0 ± 3.3	7.8 ± 5.6	0.038^*∗*^
HbA1c	8.3 ± 1.4	9.6 ± 2.6	9.9 ± 3.3	0.403
Total cholesterol	4.6 ± 1.1	4.7 ± 0.77	5.1 ± 1.0	0.12
LDL	2.7 ± 1.0	2.7 ± 0.79	3.1 ± 0.88	0.09
Ox-LDL	87.3 ± 30	117 ± 24	141.7 ± 26.6	0.048^*∗*^
CRP	3.1 ± 1.9	5 ± 3.5	7.7 ± 4.4	0.057
TAO	0.64 ± 0.3	0.61 ± 0.3	0.53 ± 0.1	0.041^*∗*^
TG	1.45 ± 0.5	1.36 ± 0.3	1.46 ± 0.4	0.675

Data is shown as mean ± standard deviation. The median difference is significant at ^*∗*^ *P* < 0.05.

**Table 4:** Correlation of different tertiles of Lp(a) with CRP, TG, TAO, and ox-LDL.

	Tertile 1		Tertile 2		Tertile 3	
	*r*	*P*	*r*	*P*	*R*	*P*
CRP	−0.33	0.42	−0.67	0.87	0.73	0.007^*∗*^
TG	0.11	0.79	0.32	0.30	0.48	0.023^*∗*^
TAO	−0.55	0.87	−0.16	0.46	−0.23	0.026^*∗*^
Ox-LDL	0.01	0.96	0.06	0.84	0.44	0.048^*∗*^

The median difference is significant at ^*∗*^ *P* < 0.05.

## Additional Points

*Limitations of the Study*. The incidence of cardiovascular disease among the participants was not assessed clinically. The smaller sample size might affect the statistical analysis of the results.
